# Electrochemical determination of glucose in beverages intended for athletes using a CuO-based nanocomposite sensor

**DOI:** 10.3389/fchem.2026.1947269

**Published:** 2026-09-14

**Authors:** Li Chao, Zhou Hongyu, Liu Baohua

**Affiliations:** School of Social Sports, Tianjin University of Sport, Tianjin, China

**Keywords:** amperometric sensor, cupric oxide, glucose sensing, graphitic carbon nitride, sport drink analysis

## Abstract

This study presents the development of a highly sensitive nonenzymatic glucose sensor based on a g-C_3_N_4_-CuO composite-modified glassy carbon electrode (GCE). CuO nanoparticles were effectively deposited on graphitic carbon nitride (g-C_3_N_4_) to fabricate the sensor. The integration of g-C_3_N_4_-CuO as a modifier significantly enhances the surface properties of the modified GCE, facilitating improved electron transfer and an increased number of active sites for glucose oxidation in NaOH solution. The electrochemical characterization of the modified electrode was performed using cyclic voltammetry (CV) and electrochemical impedance spectroscopy (EIS) techniques, revealing excellent electrochemical properties. The suggested methodology exhibited a broad calibration range from 3 to 650 µM and an impressively low detection limit of 0.5 µM. The anti-interference property was evaluated against several potential interferents, including dopamine, ascorbic acid, uric acid, L-cysteine, lactose, and sucrose, demonstrating high selectivity for glucose detection. Additionally, real sample analysis conducted on sports drinks confirmed the practical applicability and accuracy of the sensor. In addition, the sensor presented remarkable reproducibility and satisfactory storage stability. The proposed sensing platform provides a practical and promising analytical tool for nonenzymatic glucose detection in food industry applications.

## 1 Introduction

Glucose monitoring is crucial in various fields, notably in clinical diagnostics and food science. In clinical settings, accurate glucose measurement is vital for managing diabetes mellitus, a chronic condition affecting millions worldwide (Cappon et al., 2017; Villena Gonzales et al., 2019). Continuous and precise monitoring of blood glucose levels is essential for diabetes management and for preventing severe complications such as neuropathy, retinopathy, and cardiovascular diseases (Vashist, 2013; Yapanis et al., 2022). In the food industry, glucose content in products such as beverages needs to be meticulously controlled and monitored to ensure quality, safety, and compliance with nutritional labeling standards (Alp et al., 2000; Delfino et al., 2011). Therefore, there is an enduring demand for reliable and efficient glucose sensors that can cater to these diverse needs.

Traditional methods for glucose analysis, such as colorimetric assays (Zhang et al., 2021), high-performance liquid chromatography (Slimestad and Vågen, 2006), and spectrophotometry (Zaitoun, 2006), are well-established but present several practical limitations. These techniques often require elaborate sample preparation, sophisticated instrumentation, and skilled personnel, making them impractical for routine and on-site testing (Park et al., 2006). Furthermore, they can be time-consuming and expensive, limiting their widespread application (Niu et al., 2016). Sensitivity and selectivity issues also arise due to potential interference from other substances present in complex biological or food matrices, necessitating further refinement and calibration of these analytical methods.

Electrochemical sensors offer a compelling alternative for glucose analysis, addressing many of the limitations of traditional techniques (Wang, 2008; Zaidi and Shin, 2016). While enzymatic electrochemical sensors have dominated the market due to their high specificity (Amor-Gutiérrez et al., 2022; Baruah et al., 2021), nonenzymatic sensors are emerging as a promising alternative (Wang et al., 2013). Enzymatic sensors rely on glucose oxidase, an enzyme that catalyzes the oxidation of glucose, producing a measurable current (Hassan et al., 2021). However, these sensors face challenges such as enzyme instability, limited operational lifespan, and susceptibility to environmental conditions like temperature and pH variations (Rinken et al., 1998). Nonenzymatic sensors, in contrast, leverage the direct electrochemical oxidation of glucose on the electrode surface, eliminating the need for biological components (Hwang et al., 2018). This results in enhanced stability, longer shelf life, and robustness against environmental fluctuations, making nonenzymatic sensors highly attractive for continuous and long-term glucose monitoring (Dong et al., 2021).

Numerous electrochemical glucose sensors have been investigated, employing various materials and configurations (Govindaraj et al., 2023). Common materials include noble metals such as gold and platinum, which exhibit excellent catalytic properties but are costly and prone to surface fouling (Tee et al., 2017). Carbon-based materials such as graphene and carbon nanotubes have also been explored, offering high surface area and conductivity (Zhu et al., 2012). However, they often require complex functionalization processes to achieve optimal performance (Luo et al., 2016). Despite these advancements, many reported sensors still suffer from limitations such as poor selectivity, low sensitivity, and high production costs, highlighting the need for innovative approaches to enhance sensor performance (Liu et al., 2020).

Nanomaterials have revolutionized the field of electrochemical sensing, offering unique properties that significantly improve sensor performance (Fritea et al., 2021; Zhu et al., 2015). Nanosized supports, particularly graphitic carbon nitride (g-C_3_N_4_) (Sreenivasulu et al., 2024), and metal oxide nanoparticles, especially copper oxide (CuO) (Sundar et al., 2018), have shown great promise in this regard. g-C_3_N_4_ is a two-dimensional material with a high surface area, excellent conductivity, and strong adsorption capacity, making it an ideal substrate for sensor development (Zou et al., 2018). CuO nanoparticles, on the other hand, exhibit outstanding catalytic activity, stability, and biocompatibility, further enhancing the electrochemical properties of the sensor (Singh et al., 2017). The synergistic combination of these materials can lead to significant improvements in the sensitivity, selectivity, and stability of sensors (Surender et al., 2023). The high surface area of g-C_3_N_4_ provides ample active sites for analyte adsorption, while its conductive nature ensures efficient electron transfer. CuO nanoparticles deposited on g-C_3_N_4_ enhance catalytic activity towards analyte oxidation/reduction, resulting in a higher current response and improved sensitivity (Hussain et al., 2024).

Several CuO- and g-C_3_N_4_-based non-enzymatic glucose sensors have already been reported, including CuO/g-C_3_N_4_ composite electrodes with broad linear ranges and low detection limits (Huang et al., 2018; Lotfi et al., 2021). Therefore, the novelty of the present work is not claimed on the basis of a completely unprecedented material combination. However, further development of such sensing platforms remains important in order to achieve simpler fabrication, lower operating potential, improved long-term stability, and reliable applicability in real samples relevant to food analysis. In particular, although many previously reported CuO-based non-enzymatic glucose sensors have demonstrated low detection limits, long-term stability remains an equally important practical parameter for routine analytical applications. Recent CuO-based glucose sensors have reported stability over periods of 20-30 days, indicating that further improvement in long-term performance is still relevant (Lu et al., 2025; Shao et al., 2024). In this context, the present study aims to develop a practically useful g-C_3_N_4_-CuO-modified glassy carbon electrode for non-enzymatic glucose detection and to evaluate its analytical performance in sports beverage samples. The proposed sensor operates at 0.4 V, exhibits a stable response over 45 days, and provides satisfactory selectivity, reproducibility, and real-sample applicability, highlighting its potential for food quality control and nutritional labeling.

## 2 Experimental section

### 2.1 Chemicals and instruments

High-purity melamine (99%) was obtained from Alfa Aesar. Melamine served as the precursor for the synthesis of g-C_3_N_4_. Copper acetate monohydrate, Cu(OAc)_2_·H_2_O (≥99% purity), absolute ethanol (≥99.8% purity), and sodium hydroxide (NaOH) pellets (≥98% purity) were sourced from Sigma-Aldrich. d-(+)-Glucose (≥99.5% purity) was acquired from Merck. All test solutions were prepared using deionized water (>18.4 MΩ cm) obtained from a Milli-Q water purification system. The glucose determinations were performed in 0.1 M NaOH solution.

The morphology of the synthesized materials was examined by transmission electron microscopy (TEM) using a JEOL JEM-2100 microscope. The crystalline structure of the samples was investigated by X-ray diffraction (XRD) using a Bruker D8 Advance diffractometer with Cu Kα radiation (λ = 1.5406 Å). X-ray photoelectron spectroscopy (XPS) measurements were carried out on an ESCALAB 250Xi X-ray photoelectron spectrometer to analyze the surface chemical composition and chemical states of the prepared materials. Electrochemical measurements were performed using an OrigaStat 100 electrochemical workstation in a conventional three-electrode system consisting of a modified or unmodified glassy carbon electrode (GCE, φ = 2 mm) as the working electrode, an Ag/AgCl electrode as the reference electrode, and a platinum wire as the counter electrode. Cyclic voltammetry (CV) was employed to investigate the electrochemical behavior of glucose oxidation at the electrode surface, electrochemical impedance spectroscopy (EIS) was used to evaluate the interfacial charge-transfer properties (frequency range of 100 kHz to 0.1 Hz), and chronoamperometry was applied for quantitative glucose determination under the selected working conditions.

### 2.2 Synthesis of g-C_3_N_4_-CuO composite

First, the g-C_3_N_4_ support was prepared via a thermal treatment method. For this purpose, 5 g of melamine was placed into a ceramic crucible. The crucible was then covered with a lid to limit the sublimation of melamine during the heating process. The crucible was subjected to a muffle furnace and heated at 550 °C for 4 h at a heating rate of 5 °C/min. After cooling, the yellow product was collected and then ground using a mortar and pestle to achieve a uniform particle size.

In order to fabricate the g-C_3_N_4_-CuO material, 2.0 g of Cu(OAc)_2_.H_2_O was dissolved in 50 mL of ethanol to prepare a copper precursor solution. Then, 1.0 g of the g-C_3_N_4_ support powder was added to 50 mL of pure water under sonication to ensure uniform dispersion. In the next step, the copper precursor solution was added dropwise to the g-C_3_N_4_ suspension under magnetic stirring. After 2 h, the mixture was heated at 80 °C under constant stirring to evaporate the solvent. After most of the solvent had evaporated, the semi-solid mixture was placed in an oven and dried at 100 °C for 8 h. The dried precursor was entered in a ceramic crucible and then calcined in air atmosphere for 3 h at 350 °C. Compared with previously reported hydrothermal-calcination and co-pyrolysis routes for CuO/g-C_3_N_4_-based materials (Huang et al., 2018), the present synthesis approach relies on incorporation of a copper precursor onto a preformed g-C_3_N_4_ support, followed by drying and calcination. This route offers a straight preparation strategy, although a direct comparison with alternative synthesis or sequential modification pathways was beyond the scope of the present study. The final product (g-C_3_N_4_-CuO) was collected and used for the next experiments.

### 2.3 g-C_3_N_4_-CuO/GCE fabrication

The glassy carbon electrode (GCE, φ = 2 mm) was polished with an alumina slurry (0.05 μm) on a polishing cloth, followed by rinsing with deionized water and sequential sonication in ethanol and deionized water. This cleaning process ensured the removal of any surface contaminants. A homogeneous suspension of the g-C_3_N_4_-CuO nanocomposite was prepared by dispersing 1.2 mg of the nanocomposite in 1 mL of ethanol, followed by ultrasonication for 5 min. Then, 6 μL of above suspension was casted onto the clean surface of GCE and allowed to dry at room temperature.

## 3 Results and discussion

### 3.1 Materials characterization

The morphological characteristics of the synthesized g-C_3_N_4_-CuO nanocomposite were investigated using TEM, as depicted in Figure 1A. The TEM image reveals a distinct layer-like morphology, indicative of the g-C_3_N_4_ sheets, which serve as a structural support for the composite material. These g-C_3_N_4_ nanosheets are beneficial for the dispersion and stabilization of the CuO nanoparticles. The CuO nanoparticles are clearly visible as darker, roughly spherical entities distributed across the g-C_3_N_4_ sheets. The size distribution of CuO nanoparticles, as estimated from the TEM image, falls within the range of 15 to 35 nm. This nanoscale dimension is crucial as it can significantly enhance the catalytic and electrochemical properties of modifier because of the high surface-to-volume ratio, which facilitates more active sites for chemical interactions. The structural characteristics and crystallinity of the synthesized materials are further confirmed by XRD analysis (Figure 1B). In addition, the morphological features observed in the TEM images are in good agreement with the XRD results, further supporting the successful formation of the composite.

**FIGURE 1:**
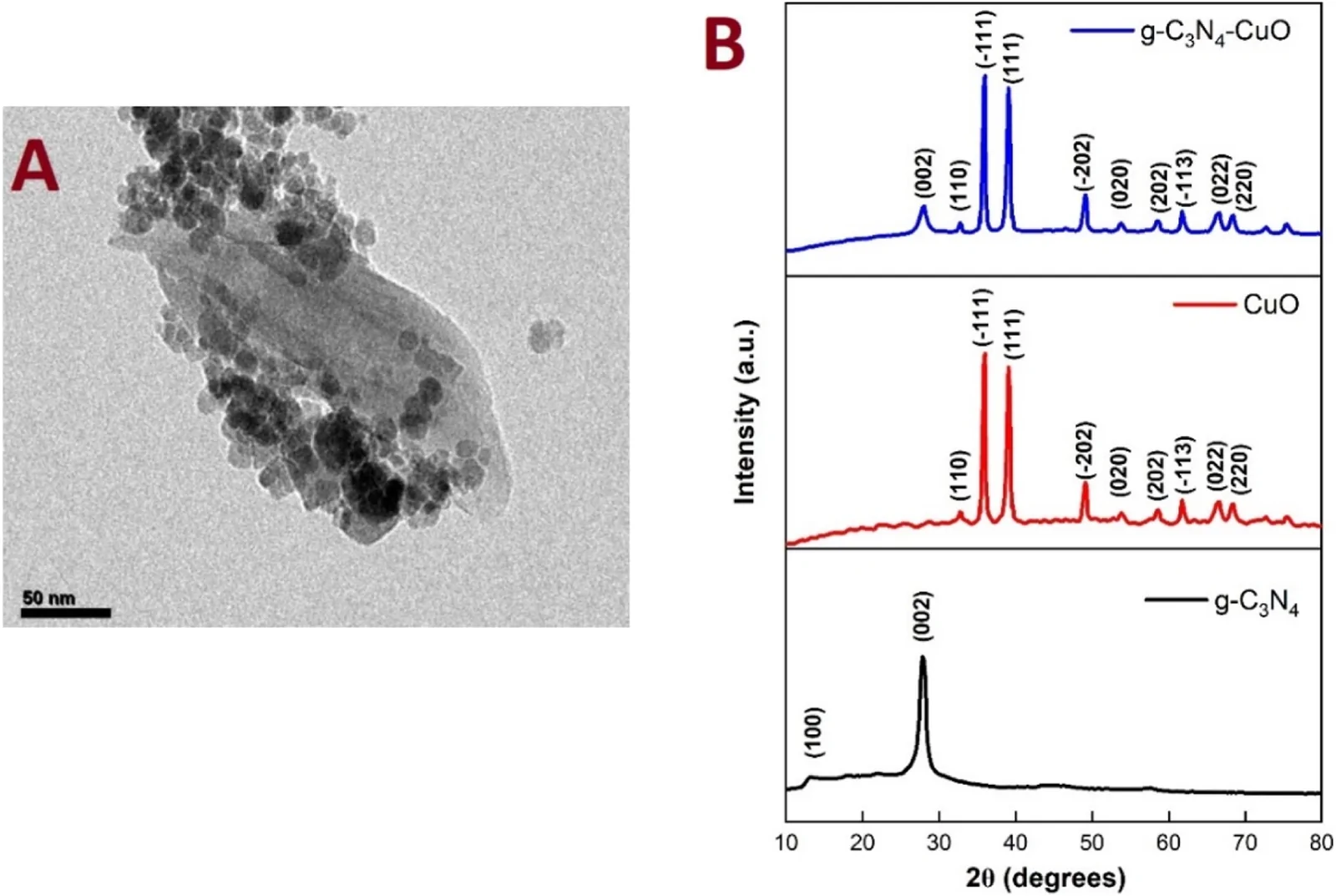
**(A)** TEM image of g-C_3_N_4_-CuO modifier. **(B)** XRD patterns of g-C_3_N_4_, CuO, and g-C_3_N_4_-CuO nanocomposite.

The XRD patterns of g-C_3_N_4_, pure CuO, and g-C_3_N_4_-CuO nanocomposite are presented in Figure 1B. The XRD pattern of g-C_3_N_4_ exhibits two characteristic diffraction peaks at 2θ ≈ 13.1° and 27.7°, corresponding to (100) and (002) planes, respectively (JCPDS No. 87-1526) (Guan et al., 2023). The pure CuO pattern shows distinct diffraction peaks at 2θ ≈ 32.5°, 35.5°, and 38.7°, which are related to (110), (−111), and (111) planes of monoclinic CuO (JCPDS No. 45-0937) (Hassani Bagheri et al., 2023). In g-C_3_N_4_-CuO composite, both sets of characteristic peaks are clearly observed, where the peaks at 13.1° and 27.7° are indexed to g-C_3_N_4_, while other peaks at 32.5°, 35.5°, and 38.7° correspond to CuO. The absence of additional impurity peaks confirms the successful formation of the g-C_3_N_4_-CuO heterostructure. The crystallite size of the CuO phase was estimated from the XRD peak broadening using the Scherrer Equation 1 (Jyoti et al., 2023):

D=Kλ/β⁡cos⁡θ (1)

where (*D*) is the crystallite size, (*K*) is the shape factor (0.9), (*λ*) is the Cu Kα wavelength (0.15406 nm), (*β*) is the full width at half maximum (FWHM) of the diffraction peak in radians, and (*θ*) is the Bragg angle.

Based on the main CuO diffraction peaks, the estimated crystallite sizes were approximately 9.2 nm for the (−111) reflection and 8.3 nm for the (111) reflection, giving an average crystallite size of approximately 8.8 nm. These values are smaller than the particle sizes observed by TEM. The difference between the XRD crystallite size and the TEM particle size is expected because the particles observed by TEM can consist of several coherently diffracting crystallites or domains.

The surface chemical composition and chemical states of the prepared materials were further investigated by XPS analysis. As shown in Figure 2a, the survey spectrum of g-C_3_N_4_-CuO nanocomposite displays the characteristic signals of C 1s, N 1s, O 1s, and Cu 2p, confirming the presence of carbon, nitrogen, oxygen, and copper in the composite. In addition, the Cu LMM Auger feature is also observed, further supporting the incorporation of copper-containing species into the hybrid structure. The high-resolution Cu 2p spectrum of g-C_3_N_4_-CuO nanocomposite (Figure 2b) exhibits two main peaks centered at 933.88 and 953.78 eV, corresponding to Cu 2p_3/2_ and Cu 2p_1/2_, respectively. The presence of the characteristic shake-up satellite peaks is indicative of Cu^2+^ species and confirms that copper is predominantly present as CuO in the nanocomposite (Ma et al., 2021). The O 1s spectrum of the g-C_3_N_4_-CuO nanocomposite (Figure 2c) can be deconvoluted into three components located at 529.68, 531.68, and 533.18 eV. The dominant peak at 529.68 eV is assigned to lattice oxygen in CuO, while the component at 531.68 eV is attributed to surface hydroxyl/defective oxygen species. The higher-binding-energy contribution at 533.18 eV is associated with weakly adsorbed oxygen-containing species or surface-adsorbed water (Alebachew et al., 2022). The high-resolution C 1s spectrum of the g-C_3_N_4_-CuO nanocomposite (Figure 2d) shows three fitted peaks at 284.68, 285.98, and 288.38 eV. The peak at 284.68 eV is assigned to adventitious/graphitic carbon, the component at 285.98 eV is related to C–N or C–NH_x_ species, and the peak at 288.38 eV is characteristic of the sp^2^-hybridized N–C=N environment in the graphitic carbon nitride framework (Morgan, 2021). The inset of Figure 2d, corresponding to pristine g-C_3_N_4_, exhibits the same characteristic carbon-nitride framework signal, indicating that the fundamental carbon skeleton of g-C_3_N_4_ is retained after CuO incorporation. Figure 2e presents the high-resolution N 1s spectrum of pristine g-C_3_N_4_ together with the much weaker N 1s signal of the g-C_3_N_4_-CuO nanocomposite in the lower part of the panel. For pristine g-C_3_N_4_, the deconvoluted peaks at 398.48, 399.28, and 400.98 eV are assigned to sp^2^-hybridized nitrogen in C–N=C units, tertiary nitrogen N–(C)_3_, and amino-type C–N–H species, respectively (Jablonski and Powell, 2017; Morgan, 2021). In contrast, the N 1s signal of the composite is markedly attenuated and is no longer clearly resolved. This does not indicate the absence of nitrogen; rather, it can be attributed to the deposition of CuO nanoparticles over the g-C_3_N_4_ surface, which partially covers the nitrogen-containing framework and suppresses the photoelectron signal from the underlying layer due to the surface-sensitive nature and limited effective attenuation length of XPS (Huang et al., 2018). Therefore, the survey spectrum still confirms the presence of nitrogen, whereas the weak high-resolution N 1s response in the composite reflects signal attenuation caused by CuO overlayer formation on g-C_3_N_4_. Overall, the XPS results confirm the successful integration of CuO with the g-C_3_N_4_ matrix.

**FIGURE 2:**
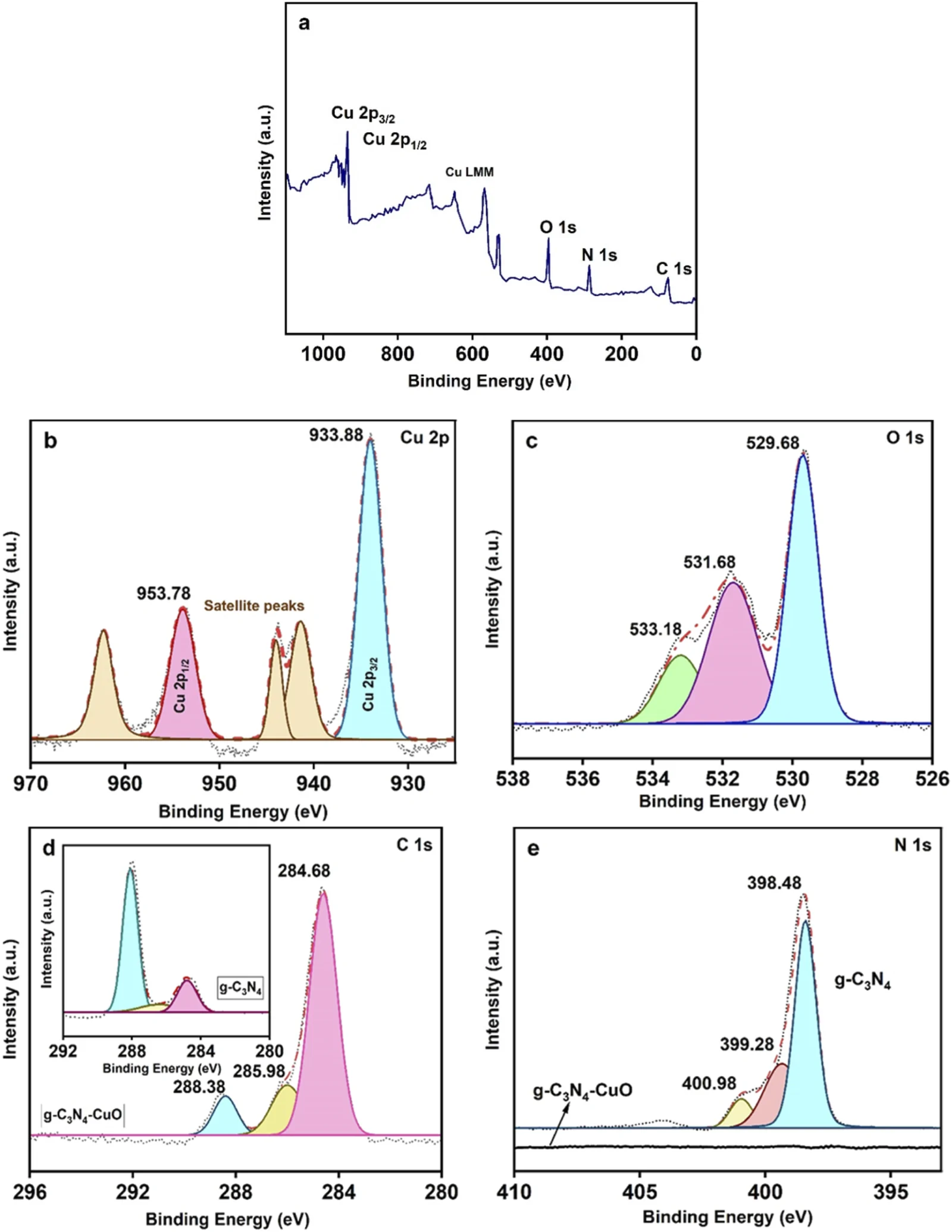
XPS spectra of g-C_3_N_4_ and g-C_3_N_4_-CuO; **(a)** survey spectrum of g-C_3_N_4_-CuO nanocomposite. **(b)** High-resolution Cu 2p spectrum of g-C_3_N_4_-CuO nanocomposite. **(c)** High-resolution O 1s spectrum of g-C_3_N_4_-CuO nanocomposite. **(d)** High-resolution C 1s spectrum of g-C_3_N_4_-CuO nanocomposite, with inset showing the corresponding C 1s spectrum of pristine g-C_3_N_4_; and. **(e)** High-resolution N 1s spectrum of pristine g-C_3_N_4_, with lower part showing the N 1s signal of g-C_3_N_4_-CuO nanocomposite.

The structural characterization results provide further insight into the origin of the enhanced electrochemical performance of the g-C_3_N_4_-CuO composite. The TEM observations indicate that CuO nanoparticles are uniformly distributed over the layered g-C_3_N_4_ nanosheets, suggesting that the g-C_3_N_4_ framework acts as an effective support that suppresses nanoparticle aggregation and helps preserve a high density of catalytically accessible CuO sites. This structural role is corroborated by the XPS analysis, which confirms the successful incorporation of CuO into the g-C_3_N_4_ matrix. In particular, the markedly attenuated N 1s signal of the composite, while still detectable in the survey spectrum, indicates partial coverage of the g-C_3_N_4_ surface by CuO nanoparticles, implying intimate interfacial contact between the two components. Such close interfacial integration is expected to promote efficient electronic communication between the conductive g-C_3_N_4_ support and the catalytically active CuO nanoparticles, thereby facilitating charge transport during the electrochemical oxidation of glucose. The beneficial influence of this heterostructure on interfacial electron-transfer kinetics is further verified by the electrochemical characterization discussed in the following section.

### 3.2 Electrochemical properties

To quantitatively compare the electroactive surface area (ECSA) of the fabricated electrodes, CV measurements were performed in a 5.0 mMK_3_ [Fe(CN)_6_]/K_4_ [Fe(CN)_6_] redox probe solution at scan rates ranging from 0.025 to 0.20 Vs^-1^. The ECSA values were calculated using the Randles–Sevcik Equation 2 (Bard et al., 2022):

$$I_{\text{pa}}=\left(2.69\times 10^{5}\right)n^{3/2}AD^{1/2}C{ʋ}^{1/2}$$ (2)

where *I*_pa_ is the anodic peak current, n is the number of electrons transferred, *A* is the electroactive surface area (cm^2^), *D* is the diffusion coefficient (cm^2^ s^-1^), *C* is the bulk concentration of the redox probe (mol cm^-3^), and *ʋ* is the scan rate (V s^-1^). The ECSA was determined from the slope of the linear relationship between the anodic peak current and the square root of the scan rate (*ʋ* ^1/2^) (Figure 3). Using *D* = 7.6 × 10^−5^ cm^2^s^-1^ and *n* = 1 for the [Fe(CN)_6_]^3-^/^4-^ redox couple, the calculated electroactive surface areas of the bare GCE, CuO/GCE, g-C_3_N_4_/GCE, and g-C_3_N_4_–CuO/GCE were 0.0148, 0.0210, 0.0251, and 0.0297 cm^2^, respectively. The progressive increase in ECSA after electrode modification confirms the successful incorporation of the nanomaterials and demonstrates that the g-C_3_N_4_–CuO composite provides the highest density of electroactive sites, thereby facilitating enhanced electron-transfer kinetics and improved electrochemical performance.

**FIGURE 3:**
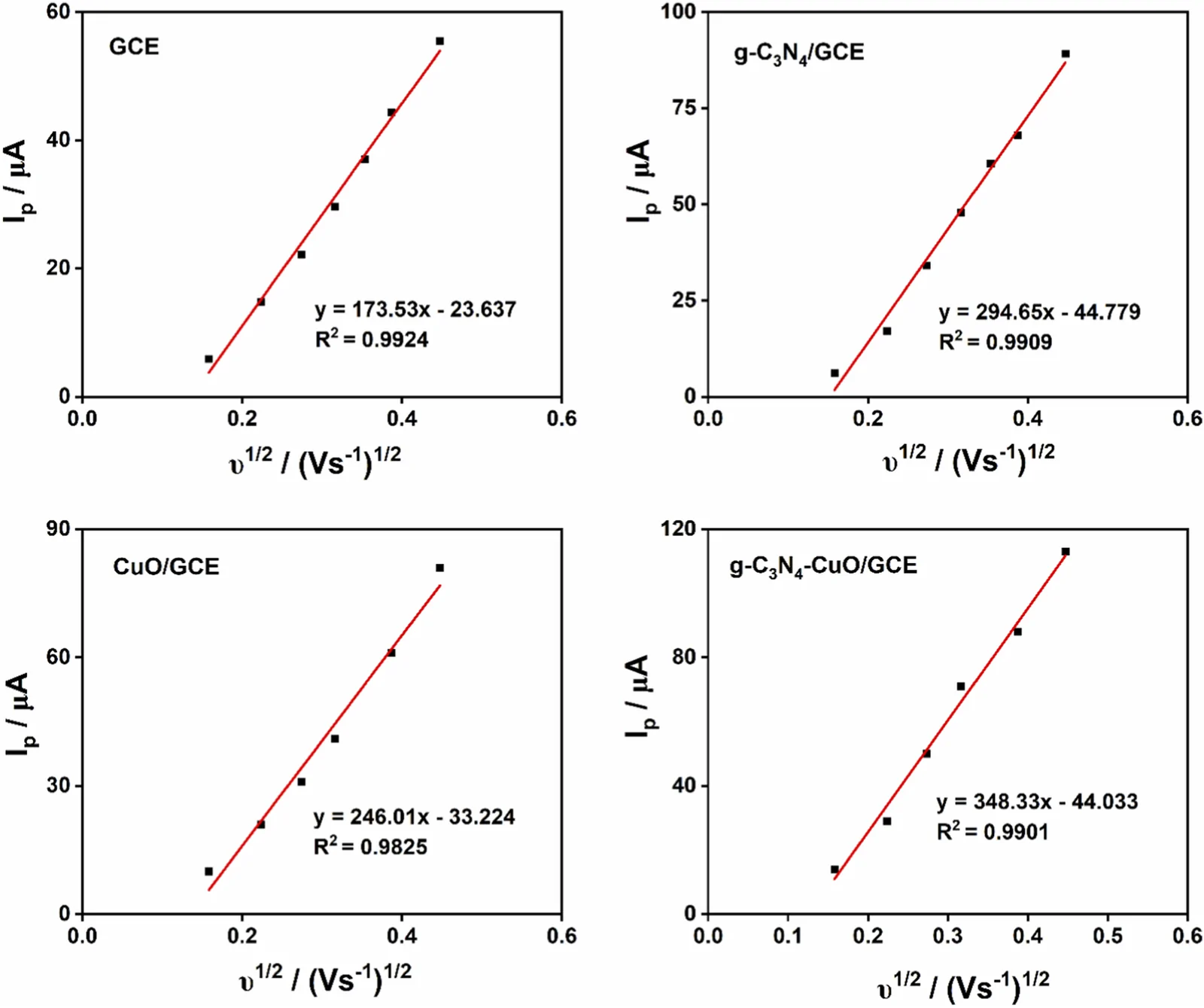
Linear relationship between the anodic peak current and the *ʋ* ^1/2^ for different electrodes.

EIS was employed at the open-circuit potential (OCP) over the selected frequency range using the Fe(CN)_6_^3-^/^4-^ redox probe solution to evaluate the interfacial electron-transfer characteristics of the fabricated electrodes. As shown in Figure 4a, the Nyquist plots of GCE, g-C_3_N_4_/GCE, CuO/GCE, and g-C_3_N_4_-CuO/GCE exhibit distinct semicircle diameters, reflecting differences in their charge-transfer behavior. The bare GCE displays the largest semicircle, whereas the g-C_3_N_4_-CuO/GCE exhibits the smallest one, indicating the most favorable interfacial electron-transfer kinetics among the investigated electrodes. To quantitatively analyze the impedance response, the experimental EIS data were fitted using the equivalent circuit *R*_s_ + [*C*_dl_ || (*R*_ct_ + *W*)], where *R*_s_, *R*_ct_, *C*_dl_, and *W* represent the solution resistance, charge-transfer resistance, double-layer capacitance, and Warburg diffusion element, respectively. The equivalent circuit adequately described the overall impedance behavior of the electrode/electrolyte interface. The fitted values of *R*_s_ remained nearly constant for all electrodes (GCE = 40.18 Ω, g-C_3_N_4_/GCE = 41.47 Ω, CuO/GCE = 40.09 Ω, and g-C_3_N_4_-CuO/GCE = 40.28 Ω), indicating that the electrolyte resistance was essentially unaffected by electrode modification. In contrast, the *R*_ct_ values decreased markedly from 179.34 Ω for the bare GCE to 120.86 Ω for g-C_3_N_4_/GCE, 88.54 Ω for CuO/GCE, and 53.37 Ω for g-C_3_N_4_-CuO/GCE.

**FIGURE 4:**
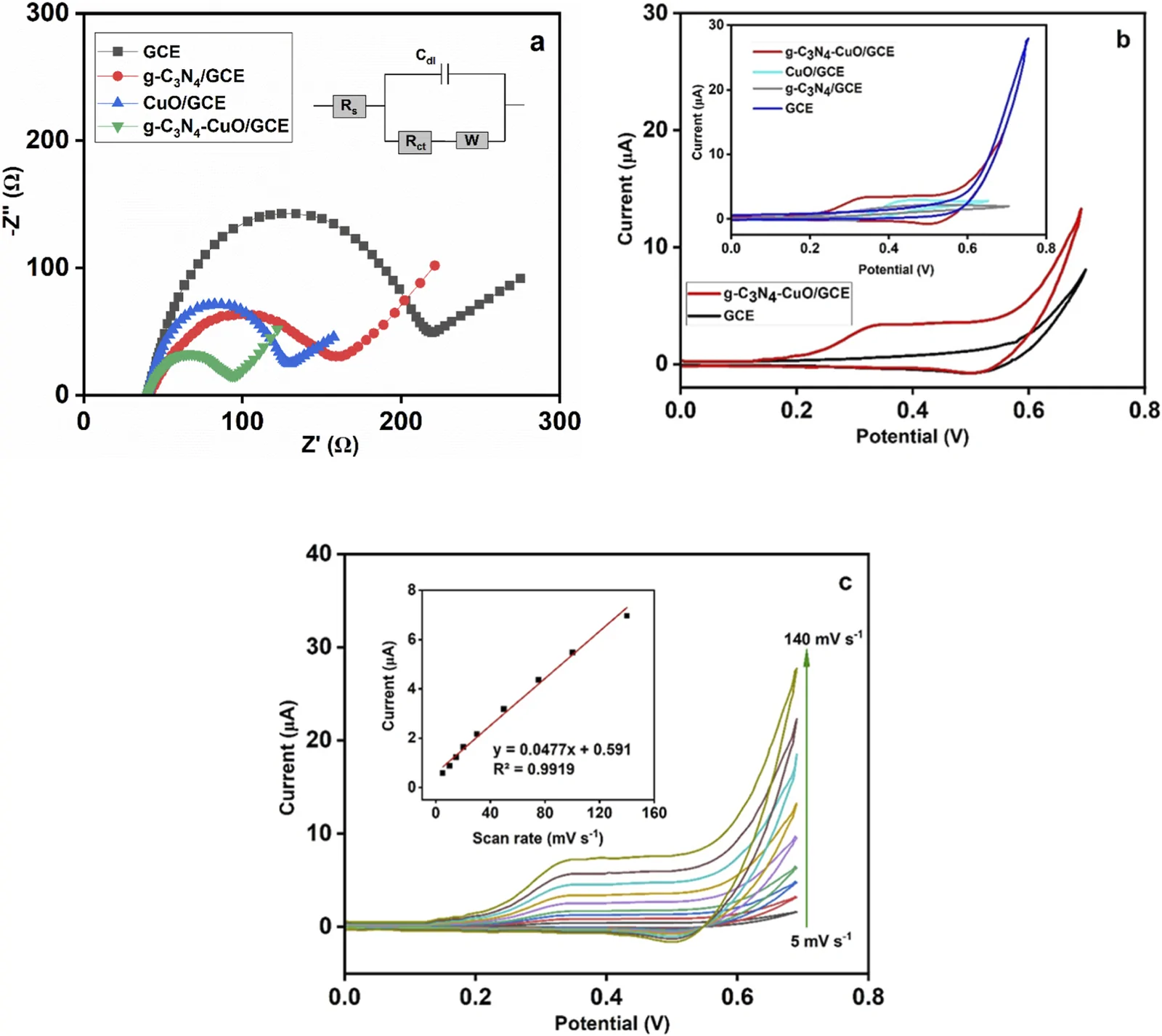
**(a)** Nyquist plots of GCE, g-C_3_N_4_/GCE, CuO/GCE, and g-C_3_N_4_-CuO/GCE recorded in the Fe(CN)_6_^3-/4-^ redox probe solution. **(b)** CV responses of GCE and g-C_3_N_4_-CuO/GCE in 0.1 M NaOH containing 100 µM glucose; inset: comparative CV curves of GCE, g-C_3_N_4_/GCE, CuO/GCE, and g-C_3_N_4_-CuO/GCE under same experimental conditions. **(c)** CV responses of g-C_3_N_4_-CuO/GCE recorded in 0.1 M NaOH containing 100 μM glucose at different scan rates (5–140 mV s^-1^); inset: corresponding linear plot of anodic peak current versus scan rate.

The substantially lower charge-transfer resistance of the g-C_3_N_4_-CuO/GCE compared with the other electrodes demonstrates that the synergistic integration of g-C_3_N_4_ and CuO facilitates interfacial electron transfer. To quantitatively evaluate the electron-transfer kinetics, the heterogeneous electron-transfer rate constant (*k*_et_) was calculated from the EIS-derived charge-transfer resistance (*R*_ct_) and the electrochemically active surface area (ECSA) using the following Equation 3 (Bhimaraya et al., 2022):

$$k_{\text{et}}=RT/n^{2}F^{2}ACR_{\text{ct}}$$ (3)

where *R* is the universal gas constant (8.314 Jmol^-1^K^−1^), *T* is the absolute temperature (*K*), n is the number of electrons transferred in the redox reaction, *F* is the Faraday constant (96,485 Cmol^-1^), *A* is the electrochemically active surface area of the electrode (cm^2^), C is the concentration of the redox species (mol cm^-3^), and *R*_ct_ is the charge-transfer resistance (Ω). The calculated ket values were 2.01 × 10^−2^, 1.75 × 10^−2^, 2.86 × 10^−2^, and 3.36 × 10^−2^ cms^-1^ for the bare GCE, g-C_3_N_4_/GCE, CuO/GCE, and g-C_3_N_4_-CuO/GCE, respectively. Notably, the g-C_3_N_4_-CuO/GCE exhibited the highest *k*_et_ value, confirming its superior electron-transfer kinetics and enhanced interfacial charge-transport capability. This quantitative improvement is consistent with the structural characteristics revealed by the TEM and XPS analyses. In particular, the intimate interfacial contact between the CuO nanoparticles and the g-C_3_N_4_ support can provide efficient pathways for charge transport while promoting a well-dispersed distribution of catalytically active CuO species. Therefore, the agreement between the EIS-derived kinetic parameters and the structural characterization strongly supports the conclusion that the enhanced electrochemical performance of the composite electrode originates from the synergistic interaction between g-C_3_N_4_ and CuO, rather than solely from an increase in the number of active sites. The corresponding *R*_ct_, ECSA, and *k*_et_ values are summarized in Table 1.

**TABLE 1:** Electrochemical parameters for different electrodes.

Electrode	R_s_ (Ω)	R_ct_ (Ω)	A (cm^2^)	k_et_ (cm s^-1^)
Bare GCE	40.18	179.34	0.0148	2.01 × 10^−2^
g-C_3_N_4_/GCE	41.47	120.86	0.0251	1.75 × 10^−2^
CuO/GCE	40.09	88.54	0.0210	2.87 × 10^−2^
g-C_3_N_4_-CuO/GCE	40.28	53.37	0.0297	3.36 × 10^−2^

The electrochemical properties of the prepared electrodes toward glucose oxidation were further investigated by CV analysis in 0.1 M NaOH containing 100 µM glucose. As shown in Figure 4b, the main panel compares CV responses of GCE and g-C_3_N_4_-CuO/GCE, while the inset presents the comparative CV curves of GCE, g-C_3_N_4_/GCE, CuO/GCE and g-C_3_N_4_-CuO/GCE under identical experimental conditions. The g-C_3_N_4_-CuO/GCE exhibited a more distinct electrochemical response in the relevant potential region than the individually modified electrodes, suggesting that the integration of g-C_3_N_4_ and CuO provides a more effective sensing interface for glucose detection. In combination with the EIS results, these findings support the favorable electrochemical behavior of g-C_3_N_4_-CuO-modified electrode.

The CV studies at various scan rates were further performed to investigate the electrochemical behavior of the g-C_3_N_4_-CuO/GCE toward glucose oxidation. As shown in Figure 4c, increase at the scan rate from 5 to 140 mV s^-1^ resulted in a gradual improvement of the anodic current response, indicating a stronger oxidation signal at higher potential scan rates. To further evaluate in dependence of oxidation response on scan rate, the anodic peak currents were plotted against the corresponding scan rates (inset of Figure 4c). A good linear relationship was obtained, following the regression equation *I* (μA) = 0.0477*ν* (mV s^-1^) + 0.591 with *R* ^2^ = 0.9919. The observed linear dependence of the oxidation current on scan rate suggests that glucose oxidation process on the g-C_3_N_4_-CuO/GCE is predominantly governed by surface-confined electrochemical behavior within the studied scan rate range. This result is consistent with the effective electrocatalytic role of the g-C_3_N_4_-CuO modifier and further supports the favorable electrochemical performance of the modified electrode toward glucose sensing (Chen et al., 2008).

### 3.3 Amperometric detection

To assess the analytical performance of the g-C_3_N_4_-CuO/GCE for nonenzymatic glucose sensing, chronoamperometric measurements were carried out at an applied potential of 0.4 V. This potential was selected based on the cyclic voltammetric behavior of glucose oxidation at the g-C_3_N_4_-CuO/GCE, since it lies in the anodic region where the glucose oxidation response becomes pronounced. Therefore, 0.4 V was used as a suitable working potential for the subsequent amperometric measurements under the investigated conditions. In addition, 0.1 M NaOH was selected as the supporting electrolyte because an alkaline medium facilitates the non-enzymatic oxidation of glucose on CuO-based electrodes. Under alkaline conditions, surface Cu(II) species are electrochemically oxidized to highly active Cu(III) oxyhydroxide species (CuOOH), which serve as the primary electrocatalytic centers for glucose oxidation. The Cu(III)/Cu(II) redox couple mediates the oxidation of glucose, leading to enhanced electron transfer and a pronounced anodic current response. Furthermore, the high concentration of OH^−^ ions promotes the formation and regeneration of these electroactive Cu(III) species, thereby improving the catalytic efficiency and analytical sensitivity of the sensor. The mechanism of glucose oxidation at the electrode surface in alkaline medium is presented in Equations 4 and 5 (Li et al., 2015; Naikoo et al., 2021). Before evaluating the final chronoamperometric response, fabrication-related parameters affecting the modifier film were further examined. Since drop-casting was used for electrode surface modification in the present work, the effects of drop-cast volume and composite concentration on sensor response were investigated. Drop-casting is widely used for direct incorporation of nanomaterials onto electrode surfaces, and the amount of deposited modifier can strongly influence film coverage, active-site accessibility, and mass/electron transport through the modifier layer. As shown in Figure 5a, the amperometric response increased with increasing drop-cast volume up to 6 μL, which can be attributed to increased surface coverage of the composite material and the availability of more electroactive sites. However, further increase in the deposited volume did not produce a significant current improvement, suggesting that sufficient surface coverage had already been reached at 6 µL. Additional loading beyond this value may mainly increase film thickness rather than the number of effectively accessible active sites. As shown in Figure 5b, the amperometric response increased with increasing composite concentration up to 1.2 mg/mL and then decreased at higher concentrations. This behavior can be attributed to the balance between the number of active sites and the formation of an overly thick or less uniform modifier layer, which may hinder analyte diffusion and interfacial electron transfer. Therefore, 6 μL and 1.2 mg mL^-1^ were selected as the fabrication conditions for the subsequent experiment.

$$\text{CuO}+{\text{OH}}^{‐}\;\to \text{ CuOOH}+e^{‐}$$ (4)

CuOOH+glucose → CuO+gluconolactone (5)

**FIGURE 5:**
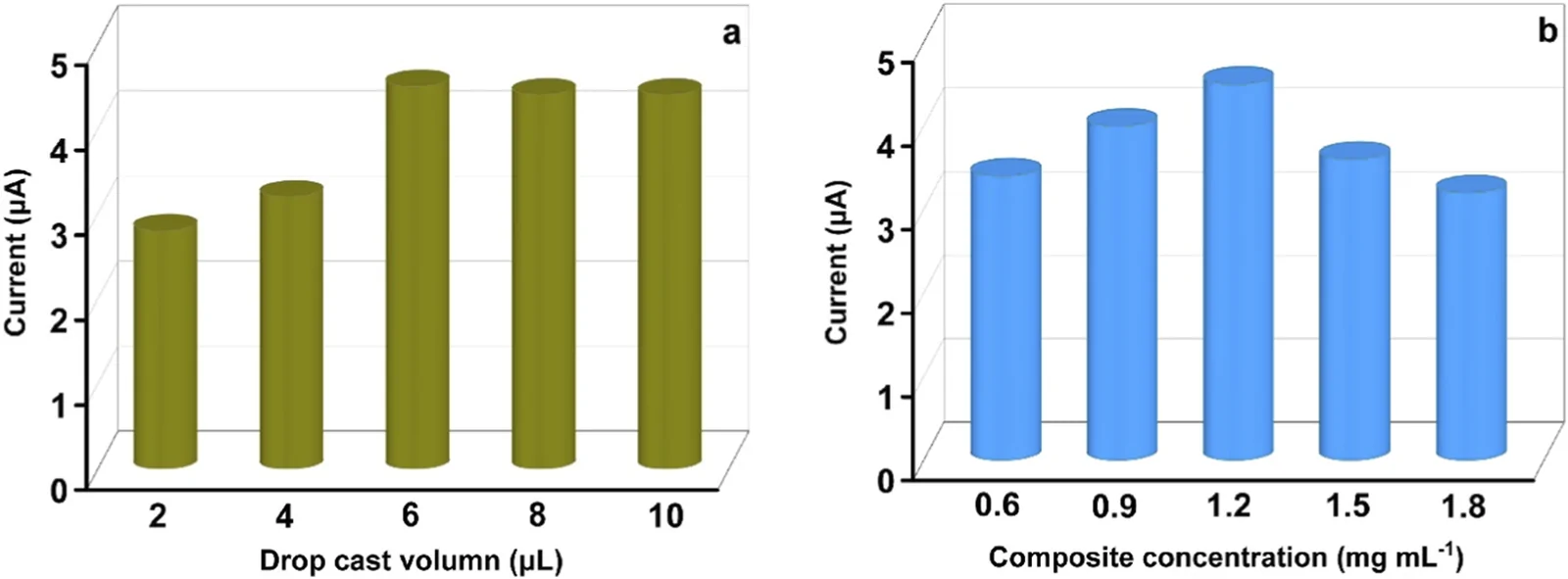
Optimization of fabrication-related parameters on amperometric response of g-C_3_N_4_-CuO/GCE toward detection of 100 µM glucose at an applied potential of 0.4 V. **(a)** Effect of drop-cast volume. **(b)** Effect of composite concentration.

Under the optimized experimental conditions, the amperometric performance of the g-C_3_N_4_-CuO/GCE toward glucose detection was further evaluated. As shown in Figure 6, successive additions of glucose produced stepwise increases in the amperometric current response of the g-C_3_N_4_-CuO/GCE at an applied potential of 0.4 V. The inset of Figure 6 presents the corresponding calibration plot, which exhibits a linear relationship between the amperometric current response and glucose concentration with a high correlation coefficient (R^2^ = 0.9987) over the concentration range of 3–650 µM. The limit of detection (LOD) was determined using the 3σ method, resulting in a value of 0.5 µM. This low LOD underscores the high sensitivity of the g-C_3_N_4_-CuO composite-based sensor, enabling the detection of trace amounts of glucose. Compared with previously reported nonenzymatic glucose sensors (Li et al., 2025; Lien et al., 2014; Ma et al., 2016; Wang et al., 2016; Wang et al., 2017; Xia et al., 2017; Zhang et al., 2015; Table 2), the proposed sensor exhibits a competitive detection limit. For a more meaningful comparison, the relevant operating conditions of the reported sensors, including the analytical method, applied potential, and supporting electrolyte, were also considered. As summarized in Table 2, these parameters vary among the reported systems and should therefore be considered when comparing their analytical performance. In the present study, glucose determination was performed by amperometry at an applied potential of 0.4 V using 0.1 M NaOH as the supporting electrolyte. Additionally, the facile synthesis of g-C_3_N_4_-CuO composite and straightforward electrode modification process contribute to the method’s practicality and potential for scalability. To evaluate the reproducibility of the sensor, we conducted analyte detection experiments using six independently prepared electrodes. The relative standard deviation (RSD) of these measurements was found to be 4.5%, indicating good reproducibility. Furthermore, the repeatability of the sensor was assessed through six successive measurements of the analyte using a single electrode, yielding an RSD of 3.0%. These low RSD values for both reproducibility and repeatability demonstrate the reliability of the proposed method, suggesting its suitability for routine glucose analysis in various applications. The stability of a nonenzymatic glucose sensor is a critical parameter for its practical applications. Subsequently, the long-term stability of g-C_3_N_4_-CuO/GCE was examined. To assess the stability of the sensor, the amperometric response to glucose over an extended period was studied. The sensor’s response was tested repeatedly over 45 days to ensure the reliability and consistency of its performance. As illustrated in the stability plot (Figure 7a), the sensor maintained an impressive level of stability over the 45-day period. After 45 days of storage, the sensor retained 90.2% of its initial current response, corresponding to a decrease of only 9.8%. This result demonstrates the satisfactory long-term stability of the g-C_3_N_4_-CuO/GCE sensor for glucose detection. The achieved stability confirmed the potential of the established sensor for reliable and continuous glucose monitoring in practical uses.

**FIGURE 6:**
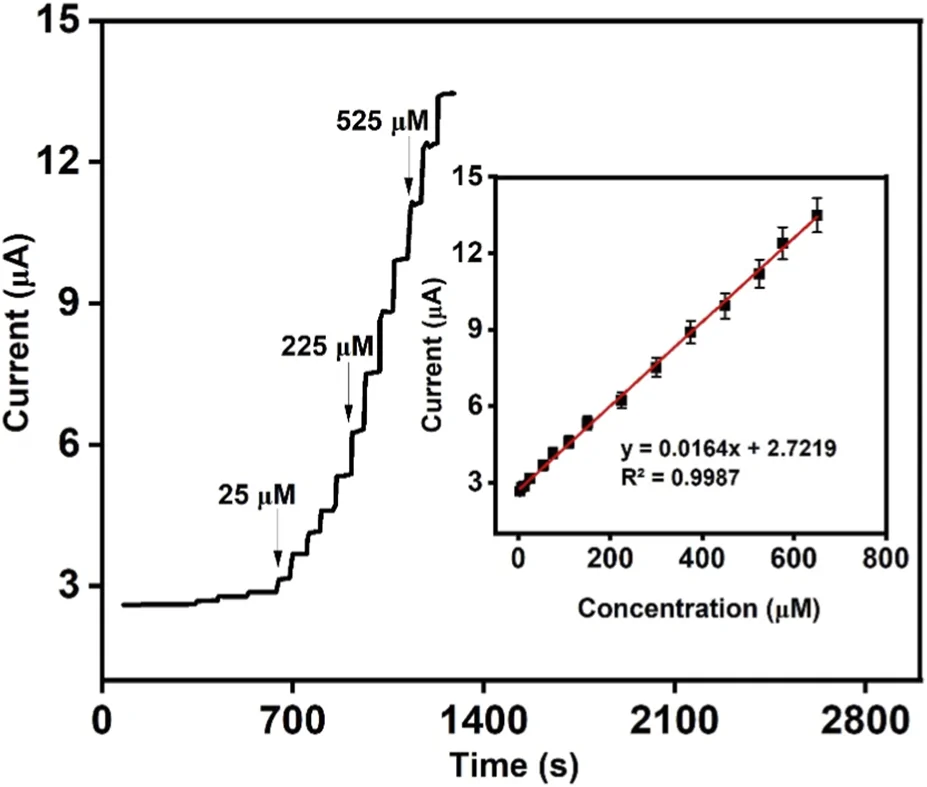
Chronoamperometric response of the g-C_3_N_4_-CuO/GCE to successive additions of glucose (3, 7, 12, 25, 55, 75, 110, 150, 225, 300, 375, 450, 525, 575, and 650 µM) at an applied potential of 0.4 V. Inset: corresponding calibration plot of amperometric current response versus glucose concentration.

**TABLE 2:** Characteristics of different glucose sensing systems.

Modifier	Linear range (mM)	Detection limit (µM)	Method	E_apply_	Electrolyte	Ref.
Co_3_O_4_ nanoclusters	0.088-7	26	Amperometry	0.5	NaOH 0.1 M	Wang et al(2017)
CNFS/Co(OH)_2_	0.01-0.12	5	Amperometry	0.55	NaOH 0.1 M	Wang et al(2016)
CoOOH nanosheet arrays	0.003-1.109	1.37	Amperometry	0.52	NaOH 0.1 M	Zhang et al(2015)
Ni(OH)_2_ flakes	0.0025-1.050	2.5	Amperometry	0.4	NaOH 0.1 M	Xia et al(2017)
Ni-Co hydroxide	0.025-3.7	-	Amperometry	0.47	NaOH 0.5 M	Lien et al(2014)
Ni-Co nanowrinkles/rGO	0.005-8.56	2	Amperometry	0.42	NaOH 0.1 M	Ma et al(2016)
Co(OH)_2_ nano-sheets	0.0015-0.46	0.8	Chronoamperometry	0.4	NaOH 0.1 M	Li et al(2025)
g-C_3_N_4_-CuO	0.003-0.65	0.5	Amperometry	0.4	NaOH 0.1 M	This study

**FIGURE 7:**
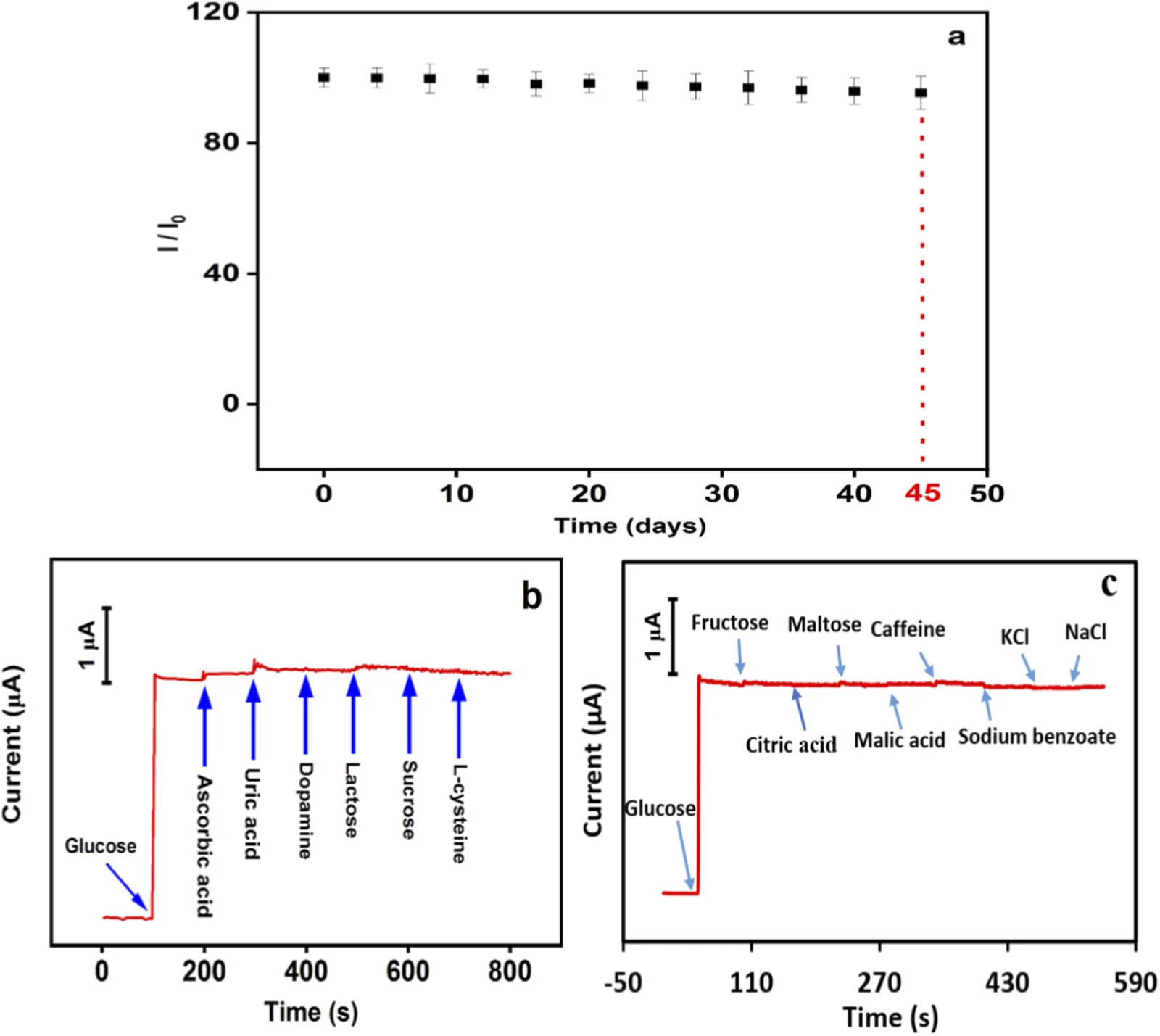
**(a)** Long-term stability of glucose sensor at ambient conditions. **(b)** Current-time curve of g-C_3_N_4_-CuO/GCE sensor upon successive additions of glucose and different common interferents. **(c)** Current-time curve of g-C_3_N_4_-CuO/GCE sensor upon successive additions of glucose and different sports-beverage-related matrix interferents.

### 3.4 Anti-interference study

The anti-interference performance of the g-C_3_N_4_-CuO/GCE toward glucose sensing was evaluated by current-time amperometry. To assess the selectivity of the proposed sensor, various potential interfering substances, including dopamine, ascorbic acid, uric acid, L-cysteine, lactose, and sucrose, were examined. These interferents were tested at concentrations 20 times higher than that of glucose. As shown in Figure 7b, the amperometric response increased markedly upon the addition of glucose, confirming the sensitivity of the sensor toward the target analyte. In contrast, the introduction of the interfering species caused no significant current change, indicating good selectivity of the proposed sensor. These results demonstrate that the g-C_3_N_4_-CuO/GCE enables selective glucose detection even in the presence of high concentrations of common interfering substances.

Considering the intended application of the sensor for glucose determination in sports beverages, additional potential matrix-related interferents commonly associated with beverage formulations were also investigated (Figure 7c). These included fructose and maltose as coexisting sugars, citric and malic acids as organic acid components, caffeine, sodium benzoate as a preservative, and KCl and NaCl as representative electrolytes. These interferents were tested at concentrations 20 times higher than that of glucose. Following the addition of glucose, the successive introduction of these beverage-related components produced no appreciable change in the steady-state current response. These results indicate that the tested beverage-related components do not significantly interfere with the amperometric determination of glucose under the experimental conditions, supporting the suitability of the proposed sensor for glucose analysis in complex sports-beverage matrices.

### 3.5 Analysis of real samples

To evaluate the practical applicability of the g-C_3_N_4_-CuO/GCE for glucose sensing, amperometric measurements were conducted in stirred solutions at an applied potential of 0.4 V. Four commercial beverages recommended for sports nutrition were selected as real samples for analysis. Sample preparation of drinks was performed according to a previously reported method (Manso et al., 2007).

Because the glucose concentrations in the commercial sports beverages were substantially higher than the linear range of the proposed sensor (3–650 μM), the samples were diluted 200-fold prior to electrochemical analysis. After dilution, the glucose concentrations were approximately 347–477 μM, placing all samples well within the established calibration range. The standard addition protocol was employed to minimize potential matrix effects associated with the complex composition of the beverages. The recovery values were calculated using the following equation: Recovery (%) = [C_found_/(C_original_ + C_added_)] × 100, where C_original_ is the glucose concentration in the original sample, C_added_ is the concentration of the added standard glucose, and C_found_ is the total glucose concentration measured after spiking (A. International, 2016). Table 3 presents the determined glucose concentrations in the drink samples, along with the corresponding recovery values. The recovery percentages ranged from 99.3% to 102.2%, indicating excellent accuracy of the proposed electrochemical sensor. The successful application of the sensor to commercial beverages demonstrates its potential for quality control in the food and beverage industry. Nevertheless, the present results are limited to the beverage formulations examined in this study. Beverages with substantially different compositions or additional untested additives may require further matrix-specific validation and appropriate sample dilution prior to analysis.

**TABLE 3:** Glucose analysis in beverages recommended for sport practice (n = 3).

Sample	Added (g/100 mL)	Found (g/100 mL)	Recovery (%)	RSD (%)
Sample 1	-	1.25 ± 0.045	-	3.6
​	0.30	1.54 ± 0.048	99.3	3.1
Sample 2	-	1.54 ± 0.065	-	4.2
​	0.30	1.88 ± 0.073	102.2	3.9
Sample 3	-	1.48 ± 0.040	-	2.7
​	0.30	1.80 ± 0.072	101.1	4.0
Sample 4	-	1.72 ± 0.065	-	3.8
​	0.30	2.01 ± 0.070	99.5	3.5

## 4 Conclusion

Although related CuO/g-C_3_N_4_-based glucose sensing systems have been previously reported, the present study demonstrates an effective g-C_3_N_4_-CuO-modified glassy carbon electrode for non-enzymatic glucose detection. The synergistic integration of CuO nanoparticles with the g-C3N4 support resulted in good electrocatalytic activity toward glucose oxidation and improved electrode surface properties. The proposed sensor operated at 0.4 V and showed a linear response over the range of 3 to 650 µM with a detection limit of 0.5 µM. Moreover, it exhibited good reproducibility, repeatability, anti-interference capability, and stable performance over 45 days. The successful application of the sensor to the determination of glucose in commercial sports beverage samples, together with satisfactory recovery values, demonstrates its practical applicability in complex matrices and its potential use in food quality control and nutritional labeling.

## 5 Declaration of generative AI and AI-assisted technologies in the writing process

During the preparation of the revised version of this work, the authors used ChatGPT to improve the readability and language of the manuscript. After using this tool, the authors reviewed and edited the content as needed and take full responsibility for the content of the published article.

## Data Availability

The raw data supporting the conclusions of this article will be made available by the authors, without undue reservation.
